# Clinical characteristics and early hematoma dynamics in a selected cohort of 199 patients with spontaneous intracerebral hemorrhage who underwent 24-hour follow-up imaging: a retrospective single-center study

**DOI:** 10.3389/fnhum.2026.1888155

**Published:** 2026-10-02

**Authors:** Pengchao Hong, Weigang Chen

**Affiliations:** Department of Neurosurgery, Dongyang People's Hospital, Dongyang, Zhejiang, China

**Keywords:** China population, early neurological deterioration, hematoma expansion, retrospective study, spontaneous intracerebral hemorrhage

## Abstract

**Background:**

Spontaneous intracerebral hemorrhage (sICH) is a devastating subtype of stroke with high morbidity and mortality. Early hematoma expansion (HE) is a key modifiable factor associated with neurological deterioration. Data on early HE dynamics in China populations with relatively late presentation (median time to admission >6 h) remain limited.

**Methods:**

This retrospective single-center study included 199 adult patients with sICH who had baseline and 24-h follow-up non-contrast computed tomography (NCCT) scans. Hematoma volume was measured using the ABC/2 method. Early HE was defined as an absolute volume increase >6 mL or a relative increase >33% on the 24-h scan. Clinical characteristics, systemic inflammatory markers (peripheral leukocyte counts), and early neurological changes (National Institutes of Health Stroke Scale [NIHSS] and Glasgow Coma Scale [GCS]) were analyzed.

**Results:**

The mean age was 58.7 ± 11.8 years, with male predominance (71.9%). Hypertension was present in 75.4% of patients. Median time from onset to admission was 10.0 h (IQR 6.0–19.5). Median initial hematoma volume was 16.2 mL (IQR 6.8–26.1). In this selected cohort of patients who survived to 24-h imaging without early surgical evacuation (median onset-to-admission 10.0 h), the observed rate of early HE was 6.0% (12/199). Because of the small number of expansion events (*n* = 12), the study was underpowered to identify baseline predictors; none of the examined demographic, clinical or radiological variables reached conventional statistical significance, although effect estimates with wide confidence intervals are provided in the main text. Peripheral leukocyte counts remained stable from admission to 24 h (*p* = 0.922). Patients with HE experienced significantly greater neurological change (median NIHSS increase 2.0 [0.0–6.5] vs. 0.0 [0.0–0.0], *p* < 0.001; median GCS change −1.0 [−2.0 to 0.0] vs. 0.0 [0.0–0.0], *p* < 0.001).

**Conclusion:**

In this selected, later-presenting single-center Chinese cohort that reached 24-h imaging without early surgery, the observed rate of early HE was low (6.0%). However, when expansion occurred, it was strongly associated with acute neurological worsening. Baseline factors did not reliably predict HE, highlighting the need for vigilant monitoring regardless of initial presentation.

## Introduction

Spontaneous intracerebral hemorrhage (ICH) is the most severe subtype of stroke, accounting for approximately 10–20% of all cerebrovascular events worldwide (Wolsink et al., 2025; Hanley et al., 2024; Sheth, 2022). It carries a particularly poor prognosis, with 30-day mortality rates ranging between 35 and 50%, and the majority of survivors left with significant long-term disability (Li et al., 2022; Fernando et al., 2021). The incidence of spontaneous ICH is substantially higher in China than in Western populations, and hypertension remains the predominant risk factor.

Early hematoma expansion (HE) is a major modifiable determinant of poor outcome in patients with spontaneous ICH (Feng et al., 2024; Chu et al., 2023). It occurs in 20–40% of cases, most frequently within the first 6–24 h after symptom onset, and is strongly associated with early neurological deterioration and increased mortality (Brouwers et al., 2014; Li et al., 2020). Despite extensive research, the precise incidence and predictors of HE show considerable variability across different populations and healthcare settings.

The role of the systemic inflammatory response, as indicated by changes in peripheral leukocyte counts, has gained increasing attention in the pathophysiology of ICH and its complications (Morotti et al., 2016). However, data regarding the early hematoma dynamics and their clinical impact in contemporary single-center Chinese cohort remain limited. The objective of this retrospective single-center study was to investigate the clinical characteristics, incidence of early hematoma expansion, and its relationship with short-term neurological outcomes in 199 patients with spontaneous intracerebral hemorrhage.

## Methods

### Study design and setting

This was a retrospective observational study conducted at a single tertiary care center. The study protocol was approved by the Institutional Review Board (IRB) of Dongyang People’s Hospital (Approval number: 2026-YX-137), and the requirement for written informed consent was waived owing to the retrospective design and use of anonymized data.

### Participants

Consecutive adult patients (≥18 years of age) diagnosed with spontaneous intracerebral hemorrhage (sICH) on non-contrast computed tomography (NCCT) who were admitted to the hospital were screened. Patients were included if they had: (1) confirmed primary sICH on baseline NCCT; (2) availability of a follow-up NCCT scan performed approximately 24 h after the baseline scan; and (3) complete clinical, laboratory, and radiological data. Between January 2022 and July 2024, 200 consecutive adult patients with spontaneous intracerebral hemorrhage on non-contrast CT who already met the imaging-follow-up criteria were identified from the source registry and assessed for eligibility. One patient was excluded because of death before the 24-h follow-up scan. The remaining 199 patients who had both baseline and approximately 24-h NCCT were included in the final analysis (see Figure 1). Patients with secondary causes of ICH, primary isolated intraventricular hemorrhage, neurosurgical intervention before the 24-h scan, or incomplete data had already been excluded upstream in the source registry; the exact numbers of these earlier exclusions could not be recovered retrospectively.

**Figure 1:**
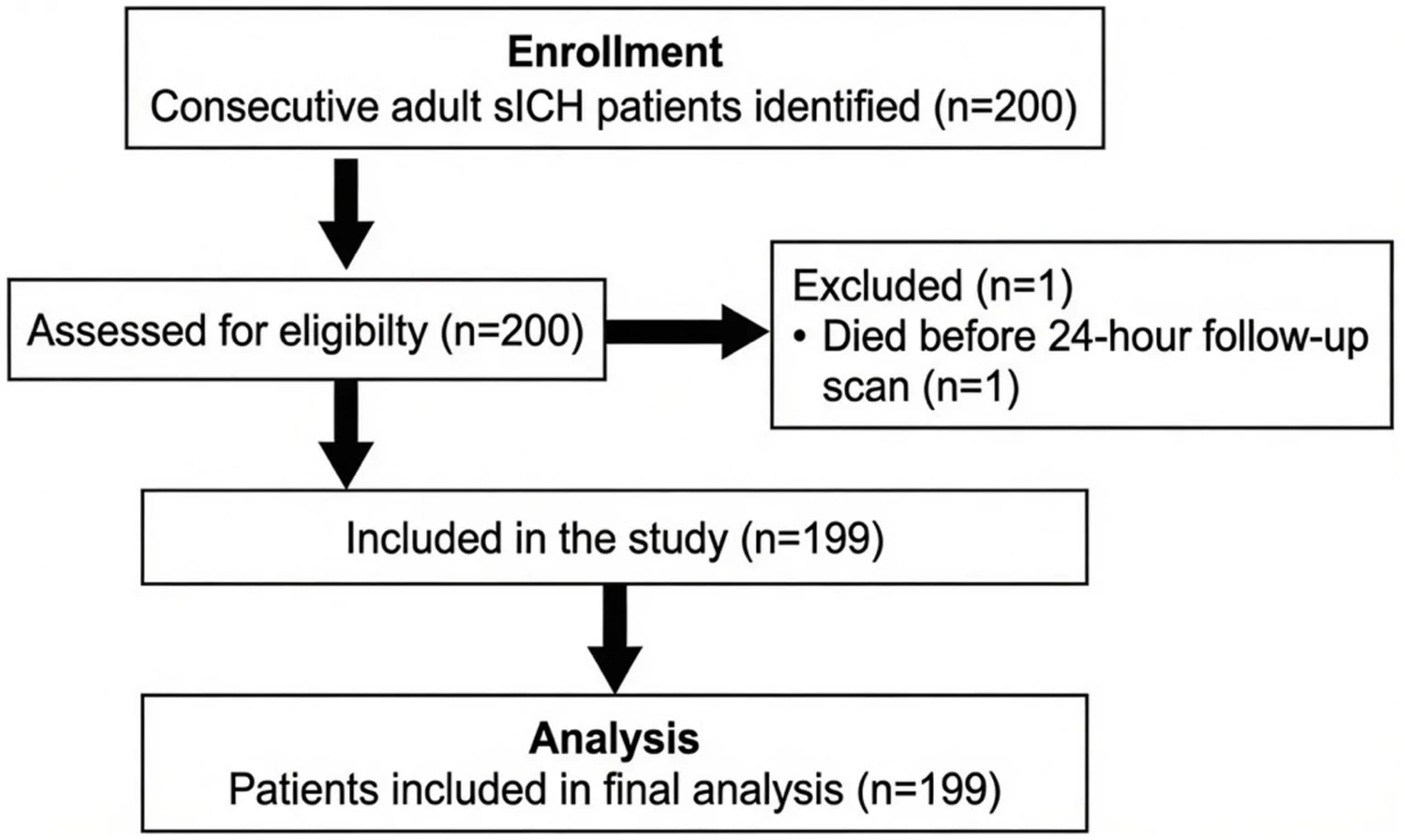
200 consecutive adult patients with spontaneous intracerebral hemorrhage who had both baseline and 24-h NCCT available were identified from the institutional registry between January 2022 and July 2024. One patient died before the follow-up scan and was excluded, leaving 199 patients in the analytic cohort. An additional, unquantified number of patients had already been excluded upstream in the source registry (secondary causes of hemorrhage, isolated primary intraventricular hemorrhage, early surgical evacuation, early death, or incomplete data). These counts could not be retrieved retrospectively. The analytic sample is therefore a selected later-presenting cohort that survived to 24-h imaging without early surgery; the true population incidence of hematoma expansion is consequently higher than the 6.0% observed here.

### Data collection

Demographic information, past medical history (hypertension, diabetes mellitus, coronary heart disease), and lifestyle risk factors (current or former smoking and alcohol consumption) were extracted from electronic medical records. Clinical presentation data included the time from symptom onset to hospital admission, admission body temperature (°C), blood glucose level (mmol/L), baseline National Institutes of Health Stroke Scale (NIHSS) score, and Glasgow Coma Scale (GCS) score. The same neurological assessments (NIHSS and GCS) were repeated at 24 h after admission. Admission blood pressure, antiplatelet or anticoagulant use, precise hematoma location (deep, lobar or infratentorial), and presence of intraventricular extension were not systematically recorded in the analytic dataset and are therefore unavailable for analysis.

Peripheral blood leukocyte (white blood cell, WBC) counts were measured upon admission and at the 24-h follow-up as part of routine laboratory evaluation to assess the early systemic inflammatory response.

Radiological Evaluation All patients underwent baseline NCCT immediately upon admission (therefore the onset-to-baseline-scan interval approximates the onset-to-admission time reported in Table 1) and a protocol-scheduled follow-up NCCT at approximately 24 h. Hematoma volumes were calculated using the ABC/2 formula on the non-contrast CT scans (Kothari et al., 1996). Early hematoma expansion (HE) was defined as an absolute volume increase of >6 mL or a relative volume increase of >33% on the 24-h follow-up scan compared with the baseline scan (Dowlatshahi et al., 2011).

**Table 1:** Baseline demographic and clinical characteristics of the study population.

Characteristic	Value (*N* = 199)
Demographics	
Age, years (mean ± SD)	58.7 ± 11.8
Male sex, *n* (%)	143 (71.9%)
Medical history and risk factors	
Hypertension, *n* (%)	150 (75.4%)
Diabetes Mellitus, *n* (%)	20 (10.1%)
Coronary Heart Disease, *n* (%)	6 (3.0%)
Current or former smoking, *n* (%)	59 (29.6%)
Alcohol consumption, *n* (%)	48 (24.1%)
Clinical presentation at admission	
Time from onset to admission, hours (median [IQR])	10.0 [6.0–19.5]
Body temperature, °C (mean ± SD)	36.8 ± 0.5
Blood glucose, mmol/L (mean ± SD)	7.6 ± 2.5
Admission NIHSS score (median [IQR])	5.0 [2.0–13.5]
Admission GCS score (median [IQR])	14.0 [11.0–15.0]

A baseline NCCT was performed on admission; onset-to-CT interval therefore approximates the onset-to-admission time.

### Clinical outcome assessment

Early neurological change was quantified as the absolute difference in NIHSS and GCS scores between admission and 24 h. These change scores are exploratory endpoints; no pre-specified dichotomous threshold (e.g., NIHSS increase ≥4 or GCS decrease ≥2) was applied. Functional outcome (modified Rankin Scale) and mortality were not available in the analytic dataset.

### Statistical analysis

Continuous variables are presented as mean ± standard deviation (SD) for normally distributed data or median with interquartile range (IQR) for non-normally distributed data. Categorical variables are presented as counts (percentages).

Between-group comparisons (HE group vs. non-HE group) for continuous variables were performed using the independent Student’s *t*-test or Mann–Whitney U test, as appropriate. Categorical variables were compared using the Pearson *χ*^2^ test or Fisher’s exact test. Paired changes in leukocyte counts between admission and 24 h were analyzed using the Wilcoxon signed-rank test. A two-sided *p*-value < 0.05 was considered statistically significant. All statistical analyses were performed using SPSS software (or equivalent statistical package). Graphical representations (violin plots with swarm overlays, kernel density plots, and box plots) were generated using GraphPad Prism.

## Results

### Baseline demographic and clinical characteristics

A total of 199 patients with spontaneous intracerebral hemorrhage who met the inclusion criteria were included in this retrospective study. The baseline demographic and clinical characteristics of the cohort are summarized in Table 1. The mean age of the study population was 58.7 ± 11.8 years (range: 29–86 years), with a clear male predominance (143 patients, 71.9%).

Regarding past medical history and lifestyle risk factors, hypertension was the most prevalent comorbidity, identified in 150 patients (75.4%). Other underlying conditions included diabetes mellitus in 20 patients (10.1%) and coronary heart disease in 6 patients (3.0%). A history of smoking and alcohol consumption was present in 29.6 and 24.1% of the patients, respectively.

Upon initial clinical presentation, the median time from symptom onset to hospital admission was 10.0 h [interquartile range (IQR): 6.0–19.5 h]. Because baseline CT was obtained on admission, the onset-to-baseline-scan interval is essentially the same as the onset-to-admission interval. Baseline physiological parameters showed a mean admission body temperature of 36.8 ± 0.5 °C and a mean admission blood glucose level of 7.6 ± 2.5 mmol/L. Neurological assessments upon admission revealed a median National Institutes of Health Stroke Scale (NIHSS) score of 5 (IQR: 2.0–13.5) and a median GCS score of 14 (IQR: 11.0–15.0), indicating that the cohort primarily consisted of patients with mild to moderate initial neurological deficits.

### Systemic inflammatory markers and physiological parameters

Systemic physiological and inflammatory responses were evaluated upon admission and at the 24-h follow-up. Upon presentation, the cohort demonstrated an average body temperature of 36.8 ± 0.5 °C and a mean admission blood glucose level of 7.6 ± 2.5 mmol/L. To assess the early systemic inflammatory response following spontaneous intracerebral hemorrhage, peripheral leukocyte (white blood cell) counts were tracked. The mean initial leukocyte count upon admission was 8.57 ± 3.08 × 10^9^/L. At the 24-h follow-up, the mean leukocyte count slightly increased to 8.69 ± 3.18 × 10^9^/L. A paired analysis revealed that this early dynamic change in circulating leukocytes within the first 24 h was not statistically significant (*p* = 0.922), indicating that the systemic inflammatory profile remained relatively stable during the hyper acute phase of the hemorrhage (Figure 2).

**Figure 2:**
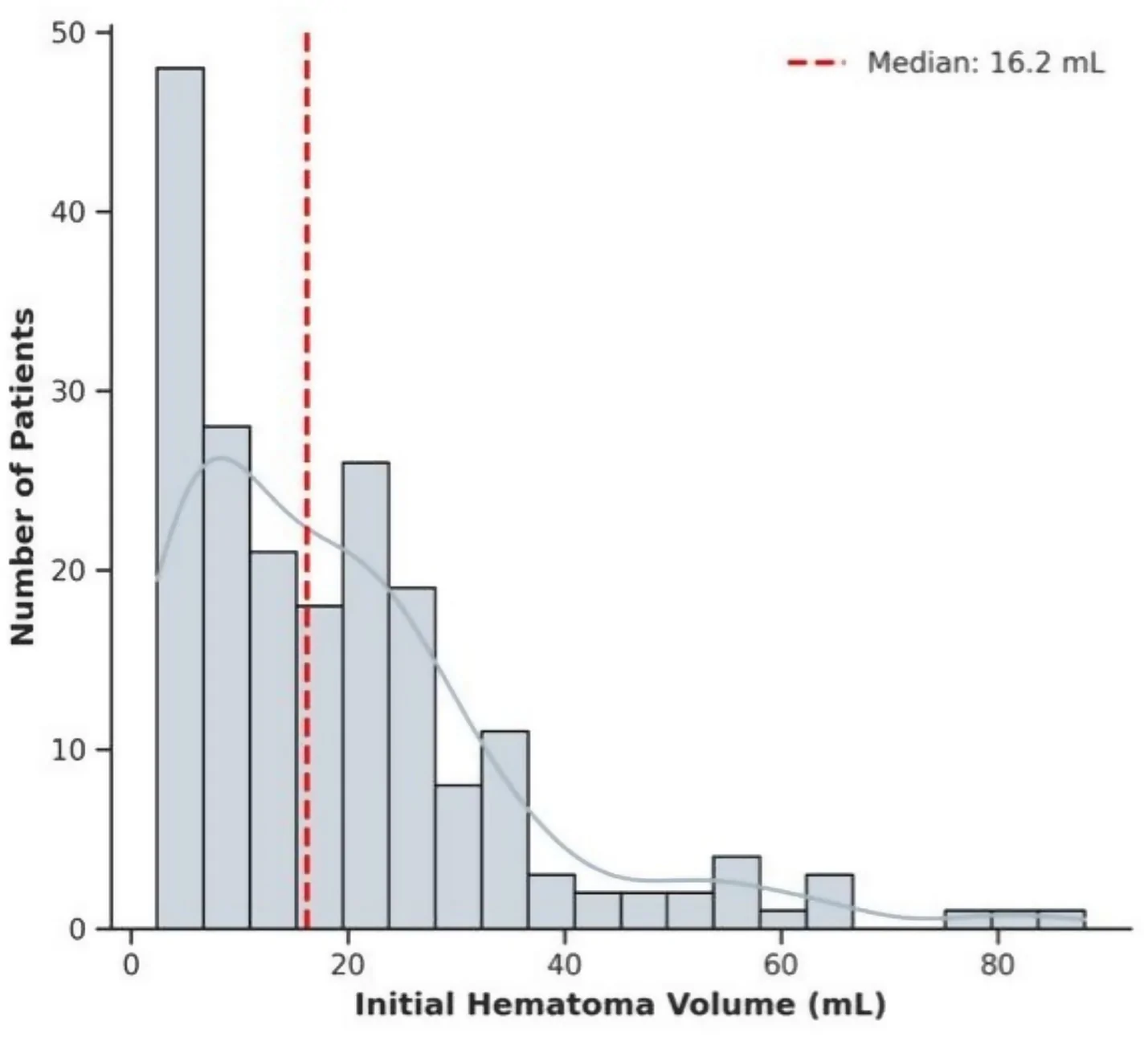
Baseline radiological features and incidence of hematoma expansion. Frequency distribution of the initial hematoma volumes for the entire patient cohort (*N* = 199) measured via non-contrast computed tomography upon admission. The curve represents the kernel density estimation, and the red dashed line indicates the median initial volume (16.2 mL).

### Baseline radiological features and incidence of hematoma expansion

Baseline NCCT scans were analyzed to determine the initial hemorrhage characteristics. The median initial hematoma volume, calculated using the ABC/2 method, was 16.2 mL (interquartile range: 6.8–26.1 mL). Early HE was defined as an absolute volume increase of >6 mL or a relative volume increase of >33% on the 24-h follow-up CT scan compared to the baseline scan. Based on these criteria, early hematoma expansion was observed in 12 patients, representing an incidence rate of 6.0% within the study cohort. Of the 12 patients who met the HE definition, 6 fulfilled both the absolute (>6 mL) and relative (>33%) criteria, 2 fulfilled only the absolute criterion, and 4 fulfilled only the relative criterion. Three of the four relative-only cases had baseline volumes <12 mL. The remaining 187 patients (94.0%) did not exhibit significant hematoma expansion during the hyperacute phase (Figure 2).

### Risk factors associated with hematoma expansion

Because data on blood pressure, antithrombotic use, hematoma location and intraventricular extension were unavailable, the following comparison is restricted to the variables that were systematically collected. To identify potential baseline demographic, clinical, and radiological factors associated with early hematoma expansion, a comparative bivariate analysis was performed between the HE group (*n* = 12) and the Non-HE group (*n* = 187). The detailed comparison is summarized in Table 2.

**Table 2:** Comparison of baseline clinical and radiological characteristics between HE and Non-HE groups.

Characteristic	HE group (*n* = 12)	Non-HE group (*n* = 187)	Effect estimate (95% CI)	*p*-value
Demographics and medical history				
Age, years (mean ± SD)	61.6 ± 10.8	58.5 ± 11.8	Mean difference 3.1 (−3.3 to 9.4)	0.360
Male sex, *n* (%)	9 (75.0%)	133 (71.1%)	OR 1.22 (0.32 to 4.68)	>0.99
Hypertension, *n* (%)	7 (58.3%)	143 (76.5%)	OR 0.43 (0.13 to 1.43)	0.173
Diabetes mellitus, *n* (%)	0 (0.0%)	20 (10.7%)	—	0.614
Coronary heart disease, *n* (%)	0 (0.0%)	6 (3.2%)	—	>0.99
Current or former smoking, *n* (%)	5 (41.7%)	54 (28.9%)	OR 1.76 (0.54 to 5.78)	0.344
Alcohol consumption, *n* (%)	2 (16.7%)	46 (24.6%)	OR 0.61 (0.13 to 2.90)	0.734
Admission parameters				
Time to admission, hours (median [IQR])	8.5 [5.8–16.2]	10.0 [6.0–20.0]	Median difference −1.5 (−6.0 to 6.0)	0.331
Body temperature, °C (mean ± SD)	36.6 ± 0.5	36.9 ± 0.5	Mean difference −0.21 (−0.51 to 0.10)	0.204
Blood glucose, mmol/L (mean ± SD)	7.8 ± 2.5	7.6 ± 2.5	Mean difference 0.18 (−1.29 to 1.64)	0.818
Leukocyte (WBC) count, ×10^9^/L (mean ± SD)	8.7 ± 3.1	8.6 ± 3.1	Mean difference 0.11 (−1.69 to 1.91)	0.905
Initial radiological and neurological status				
Initial hematoma volume, mL (median [IQR])	13.4 [9.9–22.9]	16.5 [7.0–26.0]	Median difference −3.1 (−9.1 to 8.9)	0.909
Admission NIHSS score (median [IQR])	10.0 [5.5–15.5]	4.0 [2.0–12.5]	Median difference 6.0 (0.5 to 12.0)	0.113
Admission GCS score (median [IQR])	12.0 [10.5–13.5]	14.0 [11.0–15.0]	Median difference −2.0 (−4.0 to 0.0)	0.099
24-h neurological change (exploratory)				
Δ NIHSS (24 h − admission) (median [IQR])	2.0 [0.0–6.5]	0.0 [0.0–0.0]	Median difference 2.0 (0.0 to 7.0)	<0.001
Δ GCS (24 h − admission) (median [IQR])	−1.0 [−2.0–0.0]	0.0 [0.0–0.0]	Median difference −1.0 (−2.0 to 0.0)	<0.001

Continuous variables are presented as mean ± SD or median [interquartile range, IQR]. Categorical variables are presented as counts (percentages). Effect estimates are mean differences for approximately normally distributed continuous variables, median differences (bootstrap 95% CI) for skewed continuous variables, and odds ratios (OR) for categorical variables. *p*-values were obtained from the independent Student’s t-test or Mann–Whitney U test for continuous variables and from the Pearson *χ*^2^ test or Fisher’s exact test for categorical variables. No missing data for the variables shown (complete-case data were an inclusion criterion).

Overall, baseline demographic characteristics and past medical histories were similarly distributed between the two groups. There were no statistically significant differences in age (61.6 ± 10.8 vs. 58.5 ± 11.8 years, *p* = 0.360) or the proportion of male patients (75.0% vs. 71.7%, *p* > 0.99) between the HE and Non-HE groups, respectively. Similarly, the prevalence of comorbidities such as hypertension (*p* = 0.173) and diabetes mellitus (*p* = 0.614), with median initial volume 13.4 mL [IQR 9.9–22.9] vs. 16.5 mL [7.0–26.0]; median difference −1.1 mL, 95% CI − 7.8 to 6.4; *p* = 0.91.

Upon hospital admission, initial physiological and neurological parameters were comparable. While the HE group exhibited slightly higher median initial NIHSS scores (10.0 vs. 4.0, *p* = 0.113) and lower median GCS scores (12.0 vs. 14.0, *p* = 0.099), these differences did not reach statistical significance in this cohort. Furthermore, initial hematoma volumes calculated from baseline CT scans were comparable between the HE and Non-HE groups (median 13.4 mL vs. 16.5 mL, *p* = 0.909). Time from symptom onset to hospital admission was also similar between those who expanded and those who remained stable (median 8.5 h vs. 10.0 h, *p* = 0.331).

### Impact of hematoma expansion on early neurological deterioration

The association between radiological hematoma dynamics and short-term clinical outcomes was evaluated by analyzing changes in neurological scales within the first 24 h of admission. Patients in the HE group experienced significantly more severe early neurological deterioration compared to the Non-HE group. Patients in the HE group experienced significantly greater neurological change. The median NIHSS increase was 2.0 [IQR 0.0–6.5] in the HE group versus 0.0 [0.0–0.0] in the Non-HE group (*p* < 0.001; mean 3.3 ± 3.5 versus 0.04 ± 0.3). The median GCS change was −1.0 [−2.0 to 0.0] versus 0.0 [0.0–0.0] (*p* < 0.001; mean −1.17 ± 1.34 versus −0.06 ± 0.74). (*p* < 0.001, Figure 3).

**Figure 3:**
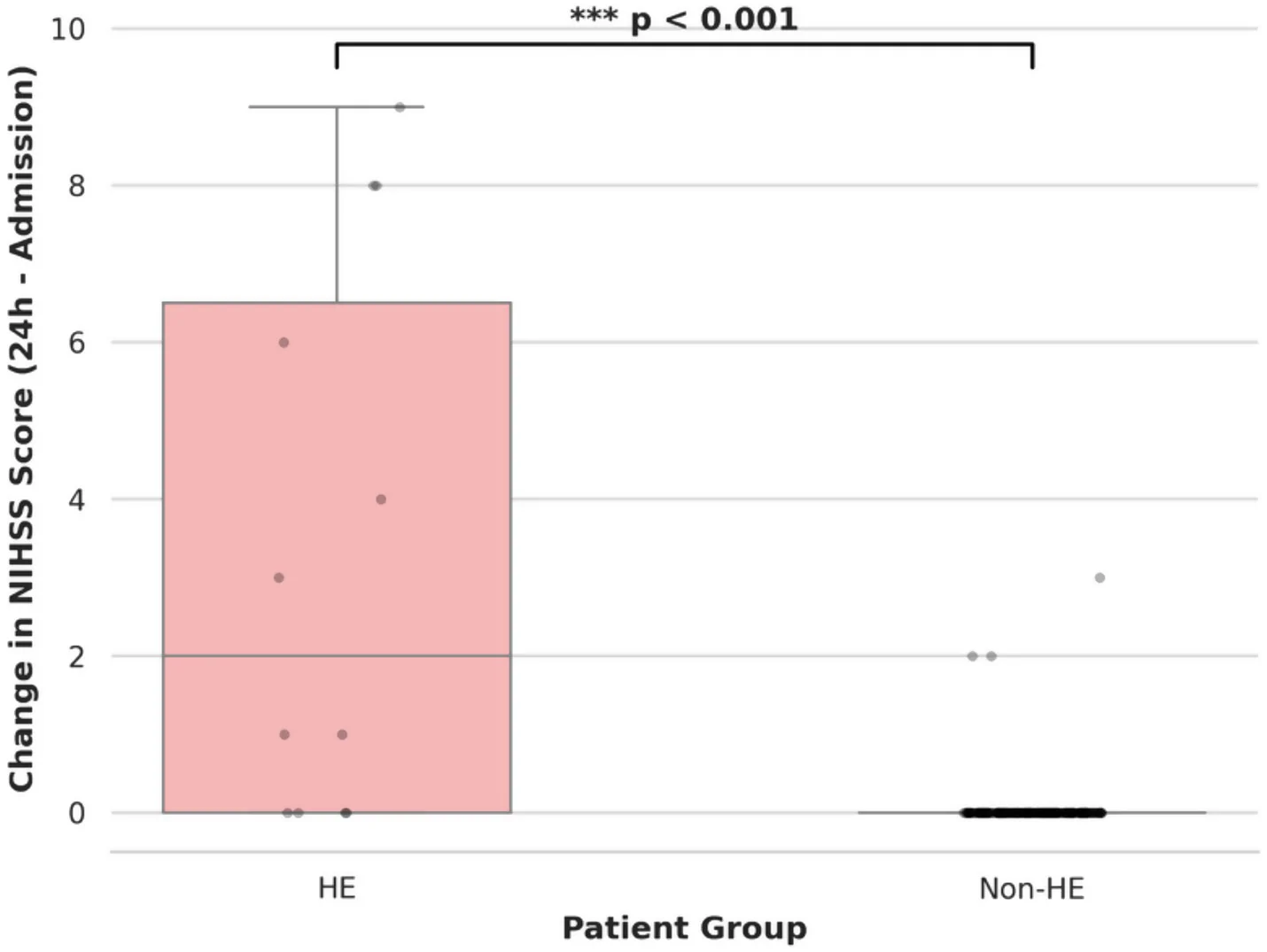
Early neurological deterioration in patients with and without hematoma expansion. The box plot displays the 24-h change in the National Institutes of Health Stroke Scale (NIHSS) scores (calculated as the 24-h score minus the admission score). Individual data points for each patient are represented by semi-transparent black dots. Patients who experienced early hematoma expansion (HE) demonstrated significantly greater neurological worsening compared to those without expansion. The horizontal lines within the boxes indicate the median, and the boundaries of the boxes represent the interquartile range (*** *p* < 0.001, Mann–Whitney U test).

Similarly, the GCS scores reflected a greater degree of clinical decline in patients with expanding hematomas. The HE group showed a mean decrease in GCS score of 1.17 ± 1.34, while the Non-HE group remained nearly constant with a mean change of −0.06 ± 0.74. These results demonstrate that even in a cohort where baseline factors did not strongly predict expansion, the occurrence of early hematoma expansion remains a critical driver of acute neurological worsening.

## Discussion

In this retrospective single-center study of 199 patients with spontaneous ICH, early hematoma expansion was observed in only 6.0% of cases. This figure must be interpreted as the incidence in a selected, later-presenting cohort that reached 24-h imaging without emergency surgical evacuation. Patients with the most rapid and extensive early growth were removed by death or surgery before the follow-up scan; the true population incidence is therefore higher (Brouwers et al., 2014; Li et al., 2020; Kazui et al., 1996).

The marked divergence from the 20–40% expansion rates reported in hyper acute cohorts is explained mainly by the timing of baseline imaging. Hematoma growth is strongly time-dependent: most volume increase occurs within the first 3–6 h after onset, after which the risk falls sharply (Kazui et al., 1996; Abou Karam et al., 2024). In the present series the median interval from symptom onset to admission (and therefore to the index CT) was 10.0 h. By that point a substantial fraction of biologically possible expansion had already occurred and was incorporated into the admission volume rather than being recorded as subsequent growth. Landmark observational studies and trials, by contrast, typically imaged patients within 3–6 h and thereby captured the period of active bleeding. A later imaging window converts what would have been classified as expansion in an ultra-early cohort into an apparently stable hematoma on the 24-h scan. This pattern is consistent with the later presentation that is common in many Chinese hospital settings.

Two further factors reduce the observed rate. First, the requirement for a protocol 24-h CT systematically excluded patients who deteriorated so rapidly that they underwent emergency evacuation or died—precisely those most likely to have expanded. Second, the analytic cohort was dominated by mild-to-moderate hemorrhages (median admission NIHSS 5, GCS 14, median volume 16.2 mL). Smaller, more stable hematomas are less likely to meet conventional absolute or relative expansion thresholds. Delayed presentation, exclusion of the sickest early expanders, and a relatively favorable severity mix therefore account for the low incidence without implying a different underlying biology of ICH in this population.

Even so, when expansion occurred it remained a potent driver of early neurological worsening. Patients with HE showed significantly greater 24-h NIHSS increase and GCS decline than those whose hematomas remained stable. This association, despite the small number of events, indicates that hematoma growth retains clinical importance after the ultra-early window has closed (Haupenthal et al., 2023).

None of the available baseline demographic, clinical or radiological variables significantly predicted expansion. The absence of identifiable predictors is most readily explained by limited statistical power (only 12 events), the attenuated risk after 6–10 h, and the restricted range of hematoma severity. Risk stratification from admission features alone is therefore particularly difficult in later-presenting patients and supports close clinical and radiological surveillance regardless of the initial appearance.

Peripheral leukocyte counts did not change appreciably between admission and 24 h and did not differ by expansion status. Although systemic inflammation has been linked to hematoma growth in some hyperacute series (Morotti et al., 2016), these data do not show a detectable early leukocyte signal in this later-presenting cohort.

A further caveat concerns measurement error of the ABC/2 method. Four of the twelve expansion events met only the relative (>33%) criterion, and three of those four had baseline volumes <12 mL. For such small hematomas a 33% relative increase corresponds to only a few millilitres, a change that can lie within the known intra and inter-observer variability of ABC/2 rather than representing true biological growth. Consequently, a fraction of the relative-only events—and therefore part of the already low 6.0% incidence—may be measurement noise. The same uncertainty applies to the association with early neurological change: if some labelled “expansions” are not genuine volume increases, the clinical contrast between groups could be either diluted or, less likely, inflated by misclassification. Planimetric or semi-automated volumetry would reduce this ambiguity in future work.

The present study has several limitations. Its retrospective, single-center design and relatively modest sample size limit statistical power and generalizability. In addition, important established correlates of hematoma expansion—admission blood pressure, antithrombotic therapy, hematoma location, and intraventricular extension—were not available, further limiting risk-factor analysis and cohort characterization. Additionally, patients who underwent early surgical intervention or died before 24-h imaging were excluded, which may have led to underestimation of HE incidence. Finally, only exploratory 24-h NIHSS and GCS change scores were available; functional outcome (mRS) and mortality data were not collected, limiting the clinical interpretation of the observed association.

In conclusion, although early HE was infrequent in this single-center Chinese cohort with delayed presentation, it remained a major driver of early neurological deterioration. These results underscore the importance of vigilant clinical and radiological monitoring in the first 24 h, even among later-presenting patients. Future larger prospective studies are needed to validate these findings and explore advanced imaging and biomarker-based prediction models in similar populations. Additionally, future studies in similar later-presenting populations should incorporate both quantitative volume measurements and qualitative NCCT markers, as well as longer-term functional outcomes.

## Data Availability

The original contributions presented in the study are included in the article/Supplementary material, further inquiries can be directed to the corresponding author.
