# Supplementary material for: Clinical characteristics and early hematoma dynamics in a selected cohort of 199 patients with spontaneous intracerebral hemorrhage who underwent 24-hour follow-up imaging: a retrospective single-center study
Source: Front Hum Neurosci. 2026 Oct 2;20:1888155. doi: 10.3389/fnhum.2026.1888155 (PMC13672531; doi:10.3389/fnhum.2026.1888155)
Supplement: Supplementary file 1 [file Supplementary_file_1.doc]

**Supplementry Figure 1. Dynamic Changes in Systemic Leukocyte Counts During the First 24 Hours.** The violin plots illustrate the distribution of peripheral WBC counts upon hospital admission and at the 24-hour follow-up for the study cohort. The width of the shaded areas corresponds to the probability density of the data at different values. Individual patient data points are overlaid as a swarm plot (black dots) to demonstrate the underlying data spread. Dashed lines within the violins indicate the median and interquartile ranges. A paired comparative analysis revealed no statistically significant dynamic change in systemic leukocyte counts between the two evaluation timepoints (ns = not significant; *p* = 0.922, Wilcoxon signed-rank test).
